# The era of the wandering mind? Twenty-first century research on self-generated mental activity

**DOI:** 10.3389/fpsyg.2013.00891

**Published:** 2013-12-18

**Authors:** Felicity Callard, Jonathan Smallwood, Johannes Golchert, Daniel S. Margulies

**Affiliations:** ^1^Centre for Medical Humanities and Department of Geography, Durham UniversityDurham, UK; ^2^Department of Social Neuroscience, Max Planck Institute for Human Cognitive and Brain SciencesLeipzig, Germany; ^3^Max Planck Research Group: Neuroanatomy and Connectivity, Max Planck Institute for Human Cognitive and Brain SciencesLeipzig, Germany

**Keywords:** mind wandering, stimulus independent thought, task-unrelated thought, daydreaming, self-generated, citation mapping, history of psychology, history of cognitive neuroscience

## Abstract

The first decade of the twenty-first century was characterized by renewed scientific interest in self-generated mental activity (activity largely generated by the individual, rather than in direct response to experimenters’ instructions or specific external sensory inputs). To understand this renewal of interest, we interrogated the peer-reviewed literature from 2003 to 2012 (i) to explore recent changes in use of terms for self-generated mental activity; (ii) to investigate changes in the topics on which mind wandering research, specifically, focuses; and (iii) to visualize co-citation communities amongst researchers working on self-generated mental activity. Our analyses demonstrated that there has been a dramatic increase in the term “mind wandering” from 2006, and a significant crossing-over of psychological investigations of mind wandering into cognitive neuroscience (particularly in relation to research on the default mode and default mode network). If our article concludes that this might, indeed, be the “era of the wandering mind,” it also calls for more explicit reflection to be given by researchers in this field to the terms they use, the topics and brain regions they focus on, and the research literatures that they implicitly foreground or ignore.

## INTRODUCTION: A NEW ERA OF MIND WANDERING RESEARCH?

One fundamental feature of the human mind is that mental activity does not cease when the mind is unoccupied by external demands. Instead, we often have thoughts and feelings that are unrelated to events in the here and now – a capacity that depends upon our mind’s ability to self-generate both cognitive and affective phenomena independently of environmental input (Smallwood, 2013). Throughout the article, we use the term “self-generated mental activity” to describe the fact that such experiences are largely generated by the individual, rather than occurring in direct response to experimenters’ instructions or to specific external sensory inputs. We use this term to describe an overarching category that encompasses a variety of phenomena – including mind wandering, daydreaming, fantasy, task-unrelated thought, and stimulus-independent thought (SIT). It should be noted that these phenomena do not exactly map on to one another (for example, self-generated mental activity would include deliberate problem-solving, and would also include daydreaming and mind-wandering, which may incorporate non-volitional processes). Despite this heterogeneity, the term “self-generated mental activity” captures a common phenomenon: that associated conscious experience is relatively more dependent on the individual’s concerns, preoccupations and hopes (i.e., self-generated), rather than immediate perceptual input (i.e., perceptually generated).

The number of studies in this special Research Topic (“Toward a psychological and neuroscientific account of the wandering mind”), coupled with the variety of topics they address, make clear that there is currently significant scientific interest in self-generated mental activity. This was not necessarily predictable: even a decade ago, investigations of these experiences were relegated to the backwaters of psychological research (see Smallwood and Schooler, 2006 for a discussion). Indeed, psychologically oriented research on self-generated mental activity was hampered for much of the twentieth century, because of the powerful influence that behaviorism exerted for many decades (e.g., Watson, 1913 and Skinner, 1953; for reflections, see Callard et al., 2012). From the 1940s to the 1970s, researchers in the field perceived that topics involving self-generated mental activity were not greeted at all positively by many senior psychologists: the field was still dominated by the pervasive meta-theory bequeathed by Watson and Skinner, which resulted in the exclusive legitimacy of behaviorist methodologies in many departments and many peer-reviewed journals (Klinger, personal communication, 2013). Nonetheless, pioneering and still influential psychological research was conducted by a small number of researchers during these decades – in particular the path-breaking research on daydreaming by Jerome Singer and his doctoral students John Antrobus and Kenneth Pope (e.g., Singer and Antrobus, 1965; Antrobus, 1968; Pope and Singer, 1978; Singer and Pope, 1978), and subsequent research by Klinger (1971), as well as by, e.g., Giambra (1974, 1993). This research was frequently not published in the most prestigious psychology journals, and often appeared in monographs (e.g., Singer, 1966, 1975) or in smaller or speciality journals (e.g., *Perceptual and Motor Skills* (e.g., Singer and Antrobus, 1963) and *Imagination, Cognition and Personality*).**

Those early works undoubtedly provided the foundations upon which isolated researchers continued to work in the late twentieth century (e.g., Einstein and McDaniel, 1997; Wegner, 1997). In this article we focus on the first decade of the twenty-first century – that moment during which research on self-generated mental activity moved out of the shadows, towards the scientific mainstream, and increasingly into journals with greater apparent scientific credibility. In contrast to earlier, widespread dismissal of or lack of interest in self-generated mental activity, many researchers now acknowledge that these phenomena have broad implications for many elements of psychological and neural function. For example, research has focused on how self-generated thought might be related to both physical (Epel et al., 2013) and mental health (Killingsworth and Gilbert, 2010); explored its implications for attentional control (Smallwood and Schooler, 2006; McVay and Kane, 2009; McVay et al., 2009; Smallwood, 2010); considered its implications for educational success (Smallwood et al., 2007a); and addressed its relation to psychiatric conditions such as depression (Smallwood et al., 2005, 2007c, 2009). A further strand of research has begun to illuminate how mind wandering is related to the nature and functions of intrinsic changes in brain activity (Mason et al., 2007; Smallwood et al., 2008; Christoff et al., 2009; Kam et al., 2011; Smallwood et al., 2013b). High-profile and/or highly cited publications in *Science* (Mason et al., 2007; Killingsworth and Gilbert, 2010) *Proceedings of the National Academy of Sciences* (*PNAS*; Christoff et al., 2009; Szpunar et al., 2013) and the *British Medical Journal* (Galera et al., 2012) make it likely that this trend will continue.

Prior to our research for this article, our sense was that “mind wandering” is now the dominant term used by researchers to characterize the self-generated mental activity in which they are interested. However, the history of psychology and proximate disciplines indicates a broader palette of terms relating to self-generated mental activity (e.g., fantasy, daydreaming; Callard et al., 2012). We were intrigued, therefore, by: (i) whether the term “mind wandering” currently does dominate research on self-generated mental activity; (ii) if it does dominate, when and how it came to do so; (iii) whether research on mind wandering, specifically, is closely related to research on other, analogous phenomena and experiences; and (iv) whether mind wandering research tends to focus on particular psychological phenomena and processes, and on particular brain functions.

Our previous research has highlighted the benefits of explicit reflection on emergent scientific fields. We have emphasized the value of analysing how certain assumptions can become (prematurely) embedded; how certain terms and concepts can become solidified over others; how normative claims (explicit or implicit) can be made about the phenomena under investigation; and how distinct bodies of research with different terminologies and ontological foundations can be brought together (or kept apart; Callard and Margulies, 2011; Callard et al., 2012; Margulies et al., 2013). This article combines our scientific and historical interests in self-generated mental activity with an exploration of how recent peer-reviewed research has described such experiences. We combine bibliometric analyses with our own expertise in the history and current state of research on self-generated mental activity. Our hope is to encourage reflection on these issues by other scientists who are building understanding of how the mind self-generates conscious experience. One central aim is to bring analytical visibility to the fact that *Frontiers* in 2013 has a special topic *on the wandering mind, *rather than, for example, on SITs, task-unrelated thoughts (TUTs) or self-generated cognition.

## METHODS AND RESULTS

We wished to investigate three different aspects of recent peer-reviewed literature that address self-generated mental activity: (i) temporal changes in a subset of terminologies used to describe self-generated mental activity; (ii) the particular research topics (e.g., particular psychological processes and/or brain functions) that characterize research currently being conducted on what we expected to be the most common category of self-generated experience: mind wandering; and (iii) where there is cross-fertilization of research interests and findings within the wide field of research on self-generated mental activity, and where there is compartmentalization of research that remains separated from other research arenas.

We determined that a combination of methods would allow us to address these three areas of inquiry: (i) we used quantitative and qualitative methods descriptively to explore recent historical changes in the use of terms for self-generated mental activity, and in the topics on which researchers investigating self-generated mental activity focus; and (ii) we performed a bibliometric analysis and visualized a citation and terminology map of the literature.

### DESCRIPTIVE ANALYSIS OF TERMINOLOGY FOR SELF-GENERATED MENTAL ACTIVITY

#### Historical changes in terminology to describe self-generated mental activity

**FIGURE 1:**
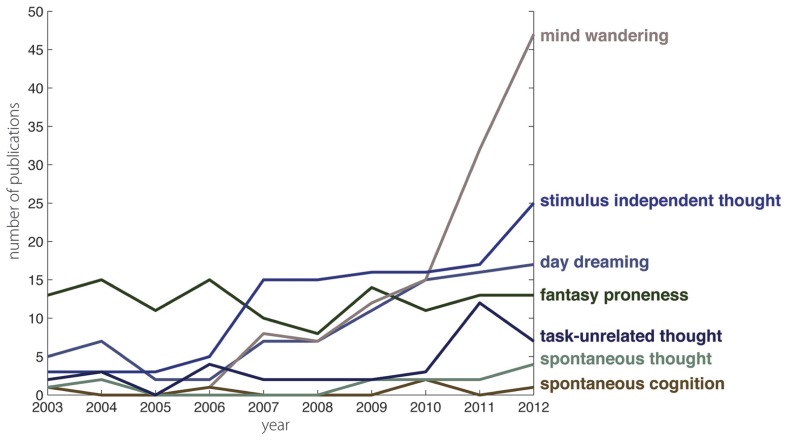
**Changes in the frequency of citations across the ten years prior to 2013**. It is apparent that the term mind wandering has seen a rapid increase in the frequency of papers using this term over this period. By contrast, some research that uses other, related terms has remained at a relatively consistent level over the same period. The *x*-axis describes the year the paper was published. The *y*-axis describes the number of papers published in each year that have the term in the title, abstract, or keywords. Different colored lines describe the different terms used to describe self-generated mental activity.

We searched the ISI Web of Science database in March 2013 in order to plot changes in terminologies used to encompass self-generated mental activity. We used our own expertise regarding historical and current research on mind wandering and related phenomena to identify the terms under which to search (these comprised: *daydreaming*, *mind wandering*, *SIT*, *TUT, spontaneous cognition, spontaneous thought, *and* fantasy proneness*). We then assembled a database of all articles that cited the three most highly cited articles for each of the terms. We did this because using the terms themselves would have restricted the pool of articles by a terminology, rather than investigating the pool of literature to which articles using those terms were contributing. We restricted our search to the decade prior to this special research topic in *Frontiers* (i.e., 2003–2012). This is a relatively short time span; however, it covers precisely that point at which we were aware that there appeared to be substantially growing interest on the part of psychologically oriented research communities in self-generated mental activity. The number of publications yielded by this procedure was then plotted over time (in relation to whether each of the original terms appeared in the title and/or abstract and/or keyword of each item in the database; **Figure 1**).

What is particularly noticeable is the difference between those terms that hover at roughly the same level throughout the decade (e.g., fantasy proneness and spontaneous cognition) and those that become increasingly prominent (daydreaming, SIT and – particularly noticeably – mind wandering). Indeed, in 2010, there is a step-change in the prominence of research that uses the term mind wandering. (Mind wandering, noticeably, does not feature until 2006, subsequent to which it very closely tracks daydreaming until the divergence in 2010 precipitated by the much larger increase in mind wandering than in daydreaming.)

#### Changes in research focus within literatures addressing mind wandering

Data from the Section “Historical changes in terminology to describe self-generated mental activity” revealed the recent and growing dominance of research using the term mind wandering. In order to interrogate this literature more closely, we investigated changes in the use of keywords in the mind wandering articles (i.e., those articles in the database using “mind wandering” in the title, abstract or keywords) to understand any changes in research focus that have accompanied the rapid growth of interest in mind wandering.

As the number of papers was relatively small (*n* = 145), and the number of keywords was relatively large (approximately 900), we reduced these terms into superordinate categories (which were based on the substantial experience of one of the authors, Jonathan Smallwood, in mind wandering research). This was performed simply to reduce the number of categories to a manageable number. Based on an analysis of the distribution of these keywords, Jonathan Smallwood identified a broad set of categories of research (*n* = 15) that accounted for a large percentage of the terms used. These categories were selected on the basis of the distribution of keywords identified and largely served to reduce pseudonyms (e.g., the terms “default mode” and “default mode network” were collapsed into a single category) and to create meaningful psychological categories (e.g., recollection and working memory were collapsed into a category of memory processes). Some of these categories were labeled using categories introduced during the process (e.g., the keywords “resting state,” “gray matter,” “prefrontal cortex,” etc. were all grouped within the category “cognitive neuroscience”). A second rater (Johannes Golchert) independently assessed the same data using the set of categories produced by Jonathan Smallwood. We interrogated these data using descriptive statistics. Although these ratings were reliable, we make no claims that they reflect a *definitive* set of research categories; they simply serve to provide a smaller number of categories with which we can explore broad changes in recent research on mind wandering.

**FIGURE 2:**
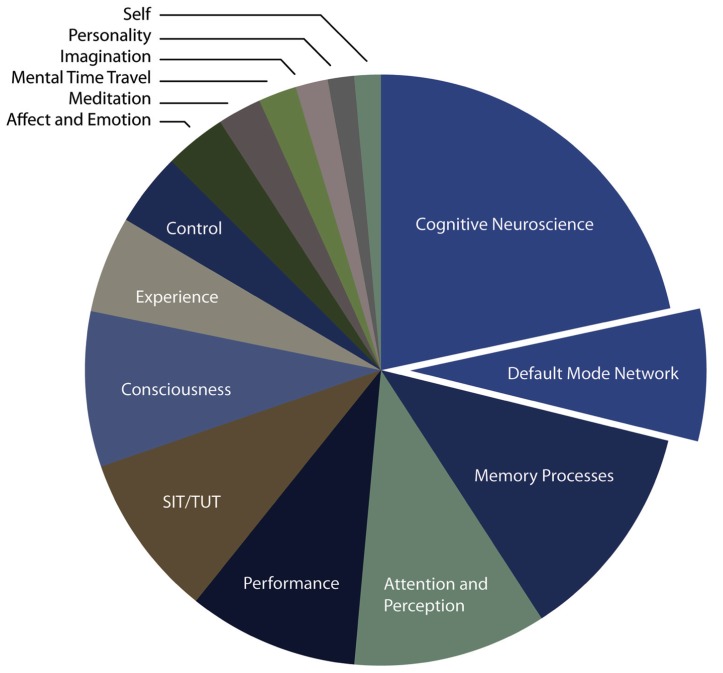
**Pie chart illustrating the different categories that form the focus of mind wandering research papers over the last decade**. The categories were identified by one author (Jonathan Smallwood), and were derived from papers’ keywords. Their applicability was confirmed by an independent assessment of these categories by a second author (Johannes Golchert). Agreement between authors was high.

We were then interested in exploring two themes. First, in order to identify the large categories of research that are associated with mind wandering over the last decade, we plotted the relative proportion of each of the categories in our sample in the form of a pie chart (**Figure 2**). The largest category of keyword associated with papers that used the term mind wandering was the term *cognitive* neuroscience, which occurred over 25% of the time (and in which the subsection “default mode network” represented a significant proportion). The next largest sets of categories were: *memory processes, attention and perception *and* performance*. Notably, therefore, approximately 25% of the key words found in our sample were related to aspects of behavior that mind wandering has been shown to derail.

**FIGURE 3:**
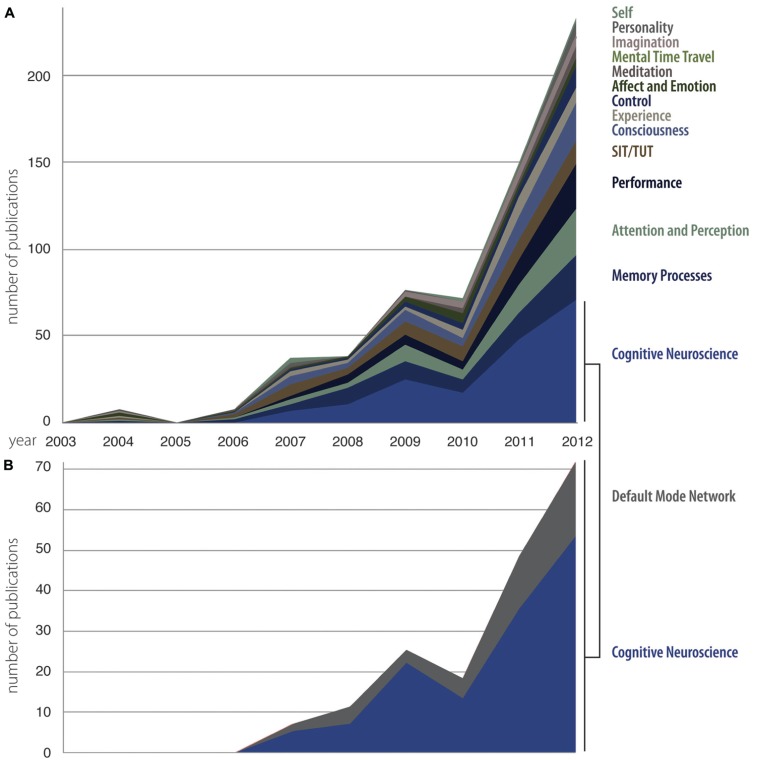
**Changes in the categories that form the focus of mind wandering research papers between 2003 and 2012.** It can be seen that almost half of the citations refer to psychological phenomena **(A)** whereas approximately the same amount refer to research focusing on cognitive neuroscience and in particular the default mode network **(B)**.

Our second aim was to consider historical trends that occurred in the use of key words over the last decade. We plotted the number of papers on mind wandering falling within each of our identified categories each year over the period of interest (**Figure 3A**). Given that the category of cognitive neuroscience accounted for over a quarter of our data, we plotted the historical trend in this category, and in one of its largest subcomponents, the default mode network, separately from all other keywords (**Figure 3B**). It can be seen that certain keywords show a pattern of slow and steady growth and are present in the majority of the years covered by our study (for example, SIT/TUT). Others have shown a rapid increase in their prevalence; of these, some were not present in the initial period (such as control), and others (such as consciousness) emerged concurrently with the turn to “mind wandering” in 2006. What is particularly noticeable is the predominance of cognitive neuroscientific research in the last 2 years of the selected period (2010–2012); within this same short time span, the visibility of research specifically on the default mode/default mode network is also striking.

### VISUALIZATION OF RESEARCH LITERATURES AND CO-CITATION NETWORKS

**FIGURE 4:**
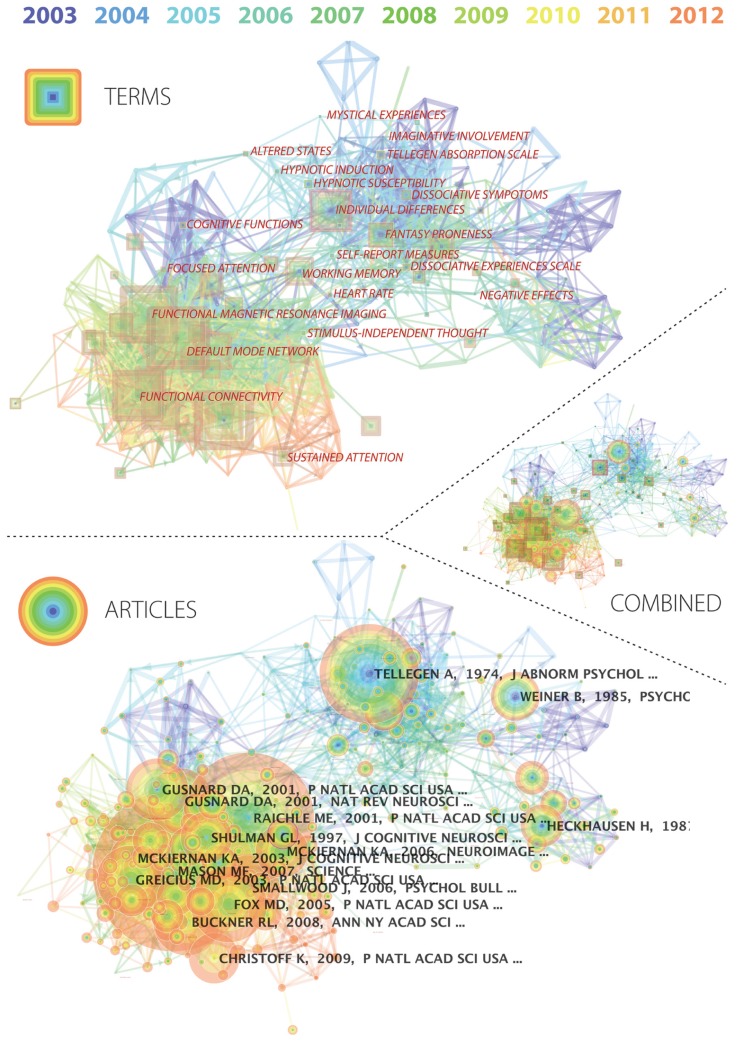
**CiteSpace was used to visualize the literature from 2003 to 2012 presented in **Figure 1****. The colors represent years, the squares are highly used terms (*top*), and the circular nodes are cited articles (*bottom*). Each colored circle represents the number of citations/uses during that year. Edge links between nodes represent co-occurrence (in the case of terms) and co-citation (in the case of articles), with the color representing the first year in which the connection was found.

The methods used above allowed us to gain a preliminary understanding of the rise of particular terminologies over the last decade, as well as the topics of enquiry being focused on within the mind wandering literature. We were also keen to have a greater understanding of the use of different terms employed in the field, the specific shifts that have occurred in the form of novel domains of investigation, and which communities cite – or do not cite – each other’s research. CiteSpace is a software tool that has been developed to map various aspects of citation networks, including the evolution of a literature over time (Chen, 2004, 2006). Based on databases of the scientific literature, it provides a flexible and interactive interface for assessing numerous aspects of the dynamics within a given field. For example, of relevance to the current analysis, CiteSpace enables the user to slice a database of literature into years of publication, and then to assess similarity of articles based on the similarity of referenced citations. Terms can also be culled from titles, keywords, and abstracts to depict their proximity based on shared inclusion in articles. The visualization platform then allows links between references or terms to depict the first year in which a connection occurred using colored edges. Subsequent usage of a term or reference can then be visualized using concentric colors that represent the frequency of citation (or use) for each year. While CiteSpace provides numerous further analytic possibilities, we constrained our analyses here (depicted in **Figure 4**) to the two analyses described above to facilitate interpretability.

The network visualization presented in **Figure 4** provides information about the organization of the citation network and use of terms over the past decade (2003–2012) using the database procedure described for the Section “Historical changes in terminology to describe self-generated mental activity” (and presented in **Figure 1**). CiteSpace deals with the problem of substantial inconsistencies in the number of articles per year by including only the most relevant, which are determined by their number of citations. The top 1% of cited articles (with a maximum of 100) from each year were included in the similarity calculations, which were based on co-citations and common term use. The proximity of nodes in the graph represents this similarity, and the visualization also reveals specific terms with high frequency of use (**Figure 4**, *top*) and articles with high accumulation of citations (**Figure 4**, *bottom*; represented by node size, where color subdivides frequency by year). The colored edges represent the earliest year in which the connection was found.

The network visualization renders immediately clear the emergence of a new tightly clustered research field beginning in 2006–2007 (green colored edges) that is characterized by terms such as “default mode network” and “functional connectivity” (**Figure 4**, *top*). “Stimulus-independent thought” falls at the edge of this cluster, closer to the psychological literature from which it emerged, though still in close proximity to the cognitive neuroscience cluster. “Working memory” is situated as a bridge between this more recent field shift toward cognitive neuroscience, and a distinct cluster described by “fantasy proneness.” On the other side of this cluster (i.e., at a distance from the cognitive neuroscience cluster) lie a circle of related terms that tie together dissociation, hypnotic susceptibility, imaginative involvement and mystical experiences. Most recently, the term “sustained attention” emerges in a tight orange cluster from 2011 from the cognitive neuroimaging literature, suggesting the emergence of a novel field of interest in self-generated mental activity.

The cited references tell another aspect of the story, indicating the role certain articles may have played in providing links between various fields. For example, Smallwood and Schooler (2006), which was published in a major psychology journal, lies right at the heart of the cognitive neuroscience cluster, depicting the central role of that article in the emerging link between “default mode” and mind wandering that has characterized the second half of the last decade. (Notably, most of the highly cited cognitive neuroscience publications (**Figure 4**, *bottom*) address the default mode and/or default mode network, e.g., Shulman et al., 1997; Gusnard et al., 2001; Raichle et al., 2001; Greicius et al., 2003; Fox et al., 2005; Buckner et al., 2008). Meanwhile in the cluster that includes fantasy proneness, dissociation and hypnotic susceptibility, the Tellegen Absorption Scale (Tellegen and Atkinson, 1974) has overwhelming significance, which extends across the entire period under investigation.

## DISCUSSION

Our analyses revealed several important features of recent research on self-generated mental activity. First, there are a number of distinct terminologies and topics of research, and these are subject to differential historical changes in terms of the frequency with which they are used. Certain terms and research topics (those associated with SIT, task-unrelated thought, and mind wandering) have grown in stature over the last decade. Our initial conjecture vis-à-vis the growing prominence of the specific term mind wandering is upheld: the last decade has not only seen an increase in research on mind wandering, but has also been marked by a solidification of the use of this term over and above alternatives.

The specific term “mind wandering” becomes prominent only very recently; prior to that, it was terms such as daydreaming and task-unrelated thought that were more dominant. Indeed, it is conceivable that a paper published in 2002 by Schooler in the high-profile *Trends in Cognitive Sciences*, which focused on dissociations between experience and meta-consciousness, and which used the phrase “catching one’s mind wandering” in the abstract, helped to facilitate a shift towards the scientific community’s use of the term “mind wandering.” (Prior to 2002, there are few uses of “mind wandering” in the psychological literature; though see, amongst others, Giambra, 1989, which operationalized “daydreaming/mind wandering” through task-unrelated thoughts, and Einstein and McDaniel, 1997, which appears to be the first to use “mind wandering” in the title). In the decades prior to those we have focused on here, the most prominent psychological research on self-generated mental activity – carried out largely by Singer, Antrobus, Klinger and Giambra – privileged constructs and phenomena that included daydreams/daydreaming, fantasy and TUTs. It is possible to advance hypotheses about why the term mind wandering superseded some of these terms. For example, the construct “fantasy” could well have been regarded (by both contemporary cognitive psychologists and cognitive neuroscientists) as too closely associated with psychoanalytically inspired research, which was increasingly jettisoned from mainstream psychology as the twentieth-century proceeded. Nonetheless, more research needs to be done to more thoroughly understand why certain terms have been overtaken by others.

The rise in “mind wandering” research has been aided by its translation from cognitive psychology into cognitive neuroscience in the last half decade. Here, it has a close tie to research on the default mode network and functional connectivity. These are, notably, research fields that, like the term “mind wandering,” came to visibility in the twenty-first century (Callard and Margulies, 2011; indeed, it could be said that the mind wandering field and the resting state/default mode network fields appear to act as motors for one another – each raising new questions for the other field to answer, and each drawing interested researchers into one another’s orbit). The close link between mind wandering research and research on the default mode network arguably implies a one-to-one mapping between a kind of experience and a particular brain network, which is likely not doing justice to the varieties of self-generated mental activity, nor to the complexity of the neural processes that contribute to these heterogeneous states. Indeed, we have argued that this tight association between mind wandering and the default mode network is at least in part owing to its historical context. That cognition has been understood largely in relation to action and environmental influences meant that “mind wandering” (as the apparent opposite of such cognition) became bound to the activation of the so-called task-negative network (the default mode network). We have argued that “this apparent ‘see-sawing’ of neural activity between two widespread brain networks suggest(s) rather intuitive – and folk-psychological – distinctions between opposing psychological functions of goal-oriented cognition and spontaneous thought” (Callard et al., 2012).

We also found that certain key articles have acted to bring together psychological and neuroscientific perspectives on self-generated mental activity. For example, in 2006 Smallwood and Schooler published “The restless mind”; while this article did not use mind wandering in its title, it did have mind wandering as a keyword, and alvso contained the sentence: “By referring to this phenomenon as mind wandering, a term familiar to the lay person, we hope to elevate the status of this research into mainstream psychological thinking.” A year later, Mason et al. (2007) published their high-profile *Science* article: its title brought together mind wandering, the default mode and SIT. This continued in 2008–2010 through the publication of articles by Buckner et al. (2008), Christoff et al. (2009), and Andrews-Hanna et al. (2010), which further cemented the links between the default network and mind wandering. In 2009, Schooler and Kane, each of whom has published well-known and highly cited research on mind wandering, organized a large symposium on “Wandering Minds and Brains” at the annual meeting of the Psychonomics Society in Boston (http://www.psychonomic.org/pdfs/PS_Call_For_Symposia_B.pdf). This symposium is likely to have acted as a stimulus for additional research (and subsequently citations) on mind wandering amongst cognitive psychologists and those in related fields. Mind wandering has also played center stage to at least two commentaries in high profile journals relating to executive control (McVay and Kane, 2010) and its neural basis (Gilbert et al., 2007). In the same vein, Killingsworth and Gilbert (2010) used the term “a wandering mind” in the title of their high-profile 2010 *Science *publication. Together, this combination of high impact papers and theoretical controversies set the stage for the very rapid growth of research on mind wandering from 2010 onward.

Second, both from our analysis here and our knowledge of the longer history of research that investigates self-generated mental activity, it can be argued that there are a number of relatively discrete research communities that have investigated such activity – often under the umbrella of distinct terms. **Figure 4** indicates that this burgeoning interest in self-generated mental activity within cognitive neuroscience has been relatively distinct since the second half of the last decade from significant areas of psychological research that have addressed related processes and phenomena. The lack of relationship between these research communities needs to be understood in part via a longer history in which the concept of “fantasy proneness” was formulated in the 1980s (originally by Wilson and Barber, 1981, 1983). Fantasy proneness is often treated as a trait (cf. mind-wandering, which has also been treated as a trait, e.g., Kane et al., 2007), was shown in certain respects to be correlated with certain psychopathologies, and was in many cases separated out from research on fantasy as process (Klinger et al., 2009). Indeed, researchers working on absorption, fantasy proneness, and dissociation (where much of the focus is on the maladaptive and/or psychopathological) tend to be relatively secluded from cognitive psychologists and cognitive neuroscientists researching mind wandering and daydreaming (where much of the focus is on processes that are commonly regarded as “normal”). This raises many interesting questions about the extent to which each distinct research sub-community might be using different kinds of normative assumptions and conceptual frameworks to understand related phenomena.

## IMPLICATIONS

We suggest that the range of terms used to investigate self-generated mental activity raises an important question for future research. The literature is heterogeneous and complex, and more research is needed to understand the conceptual, methodological and phenomenological overlaps between the objects of study being investigated by these different research communities. Certain research topics have gained traction under the umbrella of mind wandering, while others might well take shape in a field focused on the investigation of fantasy or of spontaneous cognition.

Rather than regarding such trends as a passive result of collective research agendas, we contend that it would be valuable to explore the motivations and forces that provide traction for certain terms, constructs, and approaches at particular moments in time. What is to be gained by a field through turning its research toward a previously ignored phenomenon and/or construct? Do certain formulations or terms have more flexibility than others for engendering particular interdisciplinary overlaps and crossings that have recently taken place? What is gained and what is lost when researchers investigating maladaptive and/or psychopathological manifestations of phenomena are separated from other researchers focusing on other manifestations of the same (or related) phenomena, which are frequently assumed to be “normal”? What causes certain terms and formulations to be “overtaken” by others at particular historical moments and by particular scientific communities? And what are the consequences of certain research fields remaining immune to and isolated from other research fields? Would greater cross-fertilization bring new insights into each respective research community? These are all important questions to which the research community needs to devote more attention if it hopes to provide a comprehensive account of self-generated mental activity.

One general question that this line of research raises is which terms we as a discipline should use to describe the phenomena of self-generated mental activity. Terms such as “mind wandering” and “daydreaming” have attracted the attention of writers in high-profile non-peer-reviewed publications (e.g., Jarrett, 2009). Jonah Lehrer published an essay with the normatively explicit title “The virtues of daydreaming” in *The New Yorker* in 2012 (Lehrer, 2012); John Tierney published “Discovering the Virtues of a Wandering Mind” (Tierney, 2010a) and “When the Mind Wanders, Happiness Also Strays” (Tierney, 2010b) in 2010. These articles disseminated research that has in the last few years become some of the most highly cited in the field (including Killingsworth and Gilbert, 2010, as well as research by Schooler, Smallwood, and Christoff). Such non-peer-reviewed publications, by drawing on long-standing general cultural interest in daydreams and wandering minds, have undoubtedly contributed to building excitement and interest in and outside of the scientific fields. The concept of mind wandering, while highly amenable to public interest, is an umbrella term for many different aspects of cognitive experience, and is relatively poorly specified (cf. research that focuses on *specific aspects* of self-generated mental activity, e.g., certain properties of the state, such as stimulus dependence). Different theorists are interested in developing accounts of different aspects of self-generated mental activity, and disagreements can arise because theoretical accounts to describe different elements of these experiences are often seen as contradictory, when in fact they need not be (Smallwood, 2013). One important aim for a more comprehensive account of self-generated mental activity is to develop component process accounts of the different elements of the experience.

Our analysis also revealed how much mind wandering research has focused on what self-generated mental activity *interrupts*. Much mind wandering research is “negatively” driven, because of a focus on the costs of the experience, rather than on exploring the phenomenology of the underlying processes that drive the mind to self-generate experiences. One example is the role that mind wandering plays as a contributory factor to poor concurrent task performance (see for reviews, Smallwood and Schooler, 2006; Smallwood et al., 2007b; Smallwood, 2013). Recently, mind wandering has been drawn into new arenas of research, such as meditation (Mrazek et al., 2012, 2013). This research topic has perhaps developed because of research demonstrating that mind wandering has robust links to unhappiness (Smallwood et al., 2009; Killingsworth and Gilbert, 2010). Although this research is important, there are several aspects of this focus on the *costs* of mind wandering that are worthy of comment. Although self-generated mental activity can contribute to unhappiness and error, it can also be associated with creativity (Baird et al., 2012), future planning (Baird et al., 2011), and a tendency to make patient, long-term choices (Smallwood et al., 2013a). These are all important cognitive capacities, indicating that self-generated mental activity is associated not only with psychological costs. Given that self-generated mental activity is so common in daily life, and is coupled with both costs and benefits, it seems that a more nuanced view of the experience is warranted (for a discussion see Smallwood and Andrews-Hanna, 2013). Although understanding the costs that mind wandering can have in particular contexts is important, it is worth reflecting on whether this strong focus might have occluded other approaches, which do not start from a position of focusing on what the phenomenon of interest (mind wandering) interrupts or limits. One of the most self-evident facts that phenomena such as mind wandering indicate is how little we understand about how and why human minds engage in self-generated mental activity to the extent that they do.

In conclusion, our analysis highlights important disciplinary and methodological trends that have accompanied research on self-generated mental activity in the early twenty-first century. We hope to have made explicit the complex role that heterogeneous scientific communities (in their relations or non-relations with one another) can have in consolidating particular terms, methods and areas of enquiry in research on self-generated mental activity. Ultimately, such analyses may open up new approaches, as well as new connections between different research communities. If this is, indeed, the “era of the wandering mind,” it is appropriate that explicit reflection be given by mind wandering researchers to the terms they use, the topics and brain regions they focus on, the research literatures they implicitly foreground or ignore, and the research topics in which they do or do not embed their research. Such reflection will, we hope, help to resolve contradictions and impasses that currently hamper research, and accelerate the pace of research on the intriguing puzzle that self-generated mental activity poses to our research communities.

## Conflict of Interest Statement

The authors declare that the research was conducted in the absence of any commercial or financial relationships that could be construed as a potential conflict of interest.

## Author Contributions

Daniel S. Margulies conceived of the paper; Felicity Callard and Jonathan Smallwood drafted the manuscript with contributions from Johannes Golchert and Daniel S. Margulies; Johannes Golchert and Jonathan Smallwood undertook the bibliographic analyses and ratings of keywords; Daniel S. Margulies conducted the CiteSpace visualization in consultation with Felicity Callard and Jonathan Smallwood. All authors read and approved the final manuscript.

## References

[B1] Andrews-Hanna J. R. Reidler J. S. Sepulcre J. Poulin R. Buckner R. L. (2010). Functional-anatomic fractionation of the brain’s default network. *Neuron* 65 550–562 10.1016/j.neuron.2010.02.005 20188659 PMC2848443

[B2] Antrobus J. S. (1968). Information theory and stimulus-independent thought. *Br. J. Psychol.* 59 423–430 10.1111/j.2044-8295.1968.tb01157.x

[B3] Baird B. Smallwood J. Mrazek M. D. Kam J. W. Y. Franklin M. S. Schooler J. W. (2012). Inspired by distraction: mind wandering facilitates creative incubation. *Psychol. Sci.* 23 1117–1122 10.1177/0956797612446024 22941876

[B4] Baird B. Smallwood J. Schooler J. W. (2011). Back to the future: autobiographical planning and the functionality of mind-wandering. *Conscious. Cogn.* 20 1604–1611 10.1016/j.concog.2011.08.007 21917482

[B5] Buckner R. L. Andrews-Hanna J. R. Schacter D. L. (2008). “The brain’s default network – anatomy, function, and relevance to disease,” in *Year in Cognitive Neuroscience* 2008 eds Kingstone A. Miller M. B. (Malden: Wiley-Blackwell) 1–38 10.1196/annals.1440.011 18400922

[B6] Callard F. Margulies D. S. (2011). The subject at rest: novel conceptualizations of self and brain from cognitive neuroscience’s study of the ‘resting state’. *Subjectivity* 4 227–257 10.1057/sub.2011.11

[B7] Callard F. Smallwood J. Margulies D. S. (2012). Default positions: how neuroscience’s historical legacy has hampered investigation of the resting mind. *Front. psychol. *3:321. 10.3389/fpsyg.2012.00321 PMC3437462 22973252

[B8] Chen C. (2004). Searching for intellectual turning points: progressive knowledge domain visualization. *Proc. Natl. Acad. Sci. U.S.A.* 101 5303–5310 10.1073/pnas.0307513100 14724295 PMC387312

[B9] Chen C. (2006). CiteSpace II: detecting and visualizing emerging trends and transient patterns in scientific literature. *J. Am. Soc. Inf. Sci. Technol.* 57 359–377 10.1002/asi.20317

[B10] Christoff K. Gordon A. M. Smallwood J. Smith R. Schooler J. W. (2009). Experience sampling during fMRI reveals default network and executive system contributions to mind wandering. *Proc. Natl. Acad. Sci. U.S.A.* 106 8719–8724 10.1073/pnas.0900234106 19433790 PMC2689035

[B11] Einstein G. O. McDaniel M. A. (1997). Aging and mind wandering: reduced inhibition in older adults? *Exp. Aging Res*. 23 343–354 10.1080/03610739708254035 9352291

[B12] Epel E. S. Puterman E. Lin J. Blackburn E. Lazaro A. Mendes W. B. (2013). Wandering minds and aging cells. *Clin. Psychol. Sci.* 1 75–83 10.1177/2167702612460234

[B13] Fox M. D. Snyder A. Z. Vincent J. L. Corbetta M. Van Essen D. C. Raichle M. E. (2005). The human brain is intrinsically organized into dynamic, anticorrelated functional networks. *Proc. Natl. Acad. Sci. U.S.A.* 102 9673–9678 10.1073/pnas.0504136102 15976020 PMC1157105

[B14] Galera C. Orriols L. M’Bailara K. Laborey M. Contrand B. Ribereau-Gayon R. (2012). Mind wandering and driving: responsibility case-control study. *Br. Med. J.* 345 10.1136/bmj.e8105 PMC3521876 23241270

[B15] Giambra L. M. (1974). Daydreaming across the life span: late adolescent to senior citizen. *Int. J. Aging Hum. Dev.* 5 115–140 10.2190/7AEJ-T3MA-QLGD-CCF5 4430512

[B16] Giambra L. M. (1989). Task-unrelated-thought frequency as a function of age: a laboratory study. *Psychol. Aging* 4 136–143 10.1037/0882-7974.4.2.136 2789741

[B17] Giambra L. M. (1993). The influence of aging on spontaneous shifts of attention from external stimuli to the contents of consciousness. *Exp. Gerontol.* 28 485–492 10.1016/0531-5565(93)90073-M 8224044

[B18] Gilbert S. J. Dumontheil I. Simons J. S. Frith C. D. Burgess P. W. (2007). Comment on “Wandering minds: the default network and stimulus-independent thought”. *Science* 317 43 10.1126/science.1140801 17615325

[B19] Greicius M. D. Krasnow B. Reiss A. L. Menon V. (2003). Functional connectivity in the resting brain: a network analysis of the default mode hypothesis. *Proc. Natl. Acad. Sci. U.S.A.* 100 253–258 10.1073/pnas.0135058100 12506194 PMC140943

[B20] Gusnard D. A. Akbudak E. Shulman G. L. Raichle M. E. (2001). Medial prefrontal cortex and self-referential mental activity: relation to a default mode of brain function. *Proc. Natl. Acad. Sci. U.S.A.* 98 4259–4264 10.1073/pnas.071043098 11259662 PMC31213

[B21] Jarrett C. (2009). The restless brain. *Psychologist *22(pt. 10) 836–839 10.1089/brain.2011.0019

[B22] Kam J. W. Y. Dao E. Farley J. Fitzpatrick K. Smallwood J. Schooler J. W. (2011). Slow fluctuations in attentional control of sensory cortex. *J. Cogn. Neurosci.* 23 460–470 10.1162/jocn.2010.21443 20146593

[B23] Kane M. J. Brown L. H. McVay J. C. Silvia P. J. Myin-Germeys I. Kwapil T. R. (2007). For whom the mind wanders, and when – an experience-sampling study of working memory and executive control in daily life. *Psychol. Sci.* 18 614–621 10.1111/j.1467-9280.2007.01948.x 17614870

[B24] Killingsworth M. A. Gilbert D. T. (2010). A wandering mind is an unhappy mind. *Science* 330 932–932 10.1126/science.1192439 21071660

[B25] Klinger E. (1971). *Structure and Functions of Fantasy*. New York: Wiley

[B26] Klinger E. Henning V. R. Janssen J. M. (2009). Fantasy-proneness dimensionalized: dissociative component is related to psychopathology, daydreaming as such is not. *J. Res. Pers.* 43 506–510 10.1016/j.jrp.2008.12.017

[B27] Lehrer J. (2012). *The Virtues of Daydreaming*. [Online]. Available at: 2013 [accessed June 12, 2012]

[B28] Margulies D. S. Böttger J. Watanabe A. Gorgolewski K. J. (2013). Visualizing the human connectome. *NeuroImage* 80 445–461 10.1016/j.neuroimage.2013.04.111 23660027

[B29] Mason M. F. Norton M. I. Van Horn J. D. Wegner D. M. Grafton S. T. Macrae C. N. (2007). Wandering minds: the default network and stimulus-independent thought. *Science* 315 393–395 10.1126/science.1131295 17234951 PMC1821121

[B30] McVay J. C. Kane M. J. (2009). Conducting the train of thought: working memory capacity, goal neglect, and mind wandering in an executive-control task. *J. Exp. Psychol. Learn. Mem.**Cogn.* 35 196–204 10.1037/a0014104 19210090 PMC2750806

[B31] McVay J. C. Kane M. J. (2010). Does mind wandering reflect executive function or executive failure? Comment on Smallwood and Schooler (2006) and Watkins (2008). *Psychol. Bull.* 136 188–197 10.1037/a0018298 20192557 PMC2850105

[B32] McVay J. C. Kane M. J. Kwapil T. R. (2009). Tracking the train of thought from the laboratory into everyday life: an experience-sampling study of mind wandering across controlled and ecological contexts. *Psychon. Bull. Rev.* 16 857–863 10.3758/PBR.16.5.857 19815789 PMC2760023

[B33] Mrazek M. D. Franklin M. S. Phillips D. T. Baird B. Schooler J. W. (2013). Mindfulness training improves working memory capacity and GRE performance while reducing mind wandering. *Psychol. Sci.* 24 776–781 10.1177/0956797612459659 23538911

[B34] Mrazek M. D. Smallwood J. Schooler J. W. (2012). Mindfulness and mind-wandering: finding convergence through opposing constructs. *Emotion* 12 442–448 10.1037/a0026678 22309719

[B35] Pope K. S Singer J. L. E. (1978). *The Stream of Consciousness: Scientific Investigations into the Flow of Human Experience*. New York: Plenum

[B36] Raichle M. E. MacLeod A. M. Snyder A. Z. Powers W. J. Gusnard D. A. Shulman G. L. (2001). A default mode of brain function. *Proc. Natl. Acad. Sci. U.S.A.* 98 676–682 10.1073/pnas.98.2.676 11209064 PMC14647

[B37] Shulman G. L. Fiez J. A. Corbetta M. Buckner R. L. Miezin F. M. Raichle M. E. (1997). Common blood flow changes across visual tasks: II. Decreases in cerebral cortex.* J. Cogn. Neurosci.* 9 648–663 10.1162/jocn.1997.9.5.648 23965122

[B38] Singer J. L. (1966). *Daydreaming: An Introduction to the Experimental Study of Inner Experience*. New York: Crown Publishing Group/Random House

[B39] Singer J. L. (1975). *The Inner World of Daydreaming*. Oxford: Harper & Row

[B40] Singer J. L. Antrobus J. S. (1963). A factor-analytic study of daydreaming and conceptually-related cognitive and personality variables. *Percept. Mot. Skills* 17 187–209 10.2466/pms.1963.17.1.187 14045737

[B41] Singer J. L. Antrobus J. S. (1965). Eye-movements during fantasies. *Arch. Gen. Psychiatry* 12 71–76 10.1001/archpsyc.1965.01720310073009 14221693

[B42] Singer J. L Pope K. W. E. (1978). *The Power of Human Imagination: New Methods in Psychotherapy*. New York: Plenum

[B43] Skinner B. F. (1953). *Science and Human Behavior*. New York: Macmillan Co

[B44] Smallwood J. (2010). Why the global availability of mind wandering necessitates resource competition: reply to McVay and Kane (2010). *Psychol. Bull.* 136 202–207 10.1037/a0018673

[B45] Smallwood J. (2013). Distinguishing how from why the mind wanders: a process–occurrence framework for self-generated mental activity. *Psychol. Bull.* 139 519 10.1037/a0030010 23607430

[B46] Smallwood J. Andrews-Hanna J. (2013). Not all minds that wander are lost: the importance of a balanced perspective on the mind-wandering state towards a balanced perspective of the mind-wandering state. *Front. Psychol*. 4:441 10.3389/fpsyg.2013.00441 PMC3744871 23966961

[B47] Smallwood J. Fishman D. J. Schooler J. W. (2007a). Counting the cost of an absent mind: mind wandering as an underrecognized influence on educational performance. *Psychon. Bull. Rev.* 14 230–236 10.3758/BF03194057 17694906

[B48] Smallwood J. McSpadden M. Schooler J. W. (2007b). The lights are on but no one’s home: meta-awareness and the decoupling of attention when the mind wanders. *Psychon. Bull. Rev.* 14 527–533 10.3758/BF03194102 17874601

[B49] Smallwood J. O’Connor R. C. Sudbery M. V. Obonsawin M. (2007c). Mind-wandering and dysphoria. *Cogn. Emot.* 21 816–842 10.1080/02699930600911531

[B50] Smallwood J. Fitzgerald A. Miles L. K. Phillips L. H. (2009). Shifting moods, wandering minds: negative moods lead the mind to wander. *Emotion* 9 271–276 10.1037/a0014855 19348539

[B51] Smallwood J. McSpadden M. Luus B. Schooler J. (2008). Segmenting the stream of consciousness: the psychological correlates of temporal structures in the time series data of a continuous performance task. *Brain Cogn.* 66 50–56 10.1016/j.bandc.2007.05.004 17614178

[B52] Smallwood J. O’Connor R. C. Heim D. (2005). Rumination, dysphoria, and subjective experience. *Imagin. Cogn. Pers.* 24 355–367 10.2190/AE18-AD1V-YF7L-EKBX

[B53] Smallwood J. Ruby F. J. M. Singer T. (2013a). Letting go of the present: mind-wandering is associated with reduced delay discounting. *Conscious*.* Cogn.* 22 1–7 10.1016/j.concog.2012.10.007 23178292

[B54] Smallwood J. Tipper C. Brown K. Baird B. Engen H. Michaels J. R. (2013b). Escaping the here and now: evidence for a role of the default mode network in perceptually decoupled thought. *Neuroimage* 69 120–125 10.1016/j.neuroimage.2012.12.012 23261640

[B55] Smallwood J. Schooler J. W. (2006). The restless mind. *Psychol. Bull.* 132 946–958 10.1037/0033-2909.132.6.946 17073528

[B56] Szpunar K. K. Khan N. Y. Schacter D. L. (2013). Interpolated memory tests reduce mind wandering and improve learning of online lectures. *Proc. Natl. Acad. Sci. U.S.A.* 110 6313–6317 10.1073/pnas.1221764110 23576743 PMC3631699

[B57] Tellegen A. Atkinson G. (1974). Openness to absorbing and self-altering experiences (“absorption”), a trait related to hypnotic susceptibility. *J. Abnorm. Psychol.* 83 268 10.1037/h0036681 4844914

[B58] Tierney J. (2010a). Discovering the virtues of a wandering mind. *The New YorkTimes* D1, 28th June

[B59] Tierney J. (2010b). When the mind wanders, happiness also strays. *The New YorkTimes* D1, 15th November

[B60] Watson J. B. (1913). Psychology as the behaviorist views it. *Psychol. Rev.* 20 158–177 10.1037/h0074428

[B61] Wegner D. M. (1997). “Why the mind wanders,” in *Scientific Approaches to Consciousness* eds Cohen J. D. Schooler J. W. (Mahwah, NJ: Erlbaum) 295–315

[B62] Wilson S. C. Barber T. X. (1981). “Vivid fantasy and hallucinatory abilities in the life histories of excellent hypnotic subjects (“*somnambules*”): preliminary report with female subjects,” in *Imagery: Concepts, Results, and Applications* ed. Klinger E. (New York: Plenum Press) 133–149

[B63] Wilson S. C. Barber T. X. (1983). “The fantasy-prone personality: implications for understanding imagery, hypnosis, and parapsychological phenomena,” in *Imagery: Current Theory, Research, and Applications* ed. Sheikh A. A. (New York: Wiley) 340–390

